# Unraveling atherosclerosis through single-cell RNA sequencing: insights into cellular heterogeneity and disease mechanisms

**DOI:** 10.31744/einstein_journal/2025RW1780

**Published:** 2025-11-24

**Authors:** Carlos Vicente Serrano, Matheus Laterza Ribeiro, Luciana Dornfeld Bichuette, Bruna Romanelli Scarpa Matuck, Patricia Severino, Kenneth J. Gollob, João A.C. Lima, Peter Libby

**Affiliations:** 1 Hospital Israelita Albert Einstein São Paulo SP Brazil Hospital Israelita Albert Einstein, São Paulo, SP, Brazil.; 2 Johns Hopkins Baltimore Maryland United States Johns Hopkins, Baltimore, Maryland, United States.; 3 Brigham and Women's Hospital Boston Massachusetts United States Brigham and Women's Hospital, Boston, Massachusetts, United States.

**Keywords:** Atherosclerosis, Plaque, atherosclerotic, Single-cell gene expression analysis, Sequence analysis, RNA

## Abstract

Atherosclerosis remains the leading cause of mortality worldwide. The characterization of atherosclerotic lesions reveals that they represent a chronic and progressive inflammatory condition affecting the arterial wall. Despite extensive investigations, its complex pathogenesis remains incompletely understood. Single-cell RNA sequencing (scRNA-seq) has recently emerged as a transformative tool, enabling detailed analysis of the cellular composition within atherosclerotic plaques. This approach provides a more comprehensive and nuanced understanding of plaque biology. This review provides a concise overview of current scRNA-seq methodologies and highlights their applications in atherosclerosis studies to elucidate the mechanisms underlying its onset, progression, and potential therapeutic targets.

## INTRODUCTION

Atherosclerosis is the fundamental pathological process underlying various vascular diseases, including coronary heart disease, many ischemic strokes, and peripheral artery disease, which collectively affect multiple organs such as the heart, brain, and limbs. Ischemic heart disease and stroke are the leading causes of mortality globally.^(1)^ Given its widespread impact, understanding the pathogenesis of atherosclerosis is crucial.^(2)^

Extensive studies have elucidated key mechanisms driving atherosclerosis. Endothelial dysfunction, induced by altered hemodynamic forces and reduced nitric oxide bioavailability, is a critical initiating event.^(3)^ Dysfunctional endothelial cells (ECs) enable the pathological entry of circulating low-density lipoprotein (LDL) particles into the intima^(4,5)^ and recruitment of leukocytes, triggering a localized inflammatory response that drives disease progression.^(3,6)^

Monocytes infiltrate the intima and differentiate into macrophages, which are crucial in atherosclerosis progression.^(7)^ These macrophages can internalize oxidized LDL, causing foam cell formation owing to cholesterol accumulation.^(8)^ Similarly, vascular smooth muscle cells (VSMCs) can transition into foam cells under specific stimuli, further contributing to plaque formation.^(9)^ Certain subtypes of mononuclear phagocytes secrete pro-inflammatory cytokines, promote cell migration and proliferation, and recruit additional myeloid cells, exacerbating inflammation.^(10)^ In addition, other immune cells, including platelets, neutrophils, T cells, and B cells, contribute to the inflammatory microenvironment.^(9)^

As the disease progresses, macrophage apoptosis (programmed cell death) and defective efferocytosis (impaired dead cell clearance) drive the formation of necrotic cores within plaques. The plaque core is encapsulated by a fibrous cap comprising extracellular matrix (ECM) macromolecules, including interstitial collagens, which are produced by VSMCs that have migrated toward the luminal surface.^(11)^ Plaque expansion can narrow the vascular lumen, causing downstream ischemia. However, during the early phase of atherogenesis, plaques grow outward (compensatory enlargement), which permits significant accumulation of plaque within the intima without luminal encroachment. Plaques with weakened fibrous caps are prone to rupture, triggering atherothrombosis and acute ischemic events.^(12)^

Furthermore, platelets play a critical role in monocyte recruitment to atherosclerotic plaques and in regulating macrophage and smooth muscle cell phenotypes.^(13)^ Upon infiltrating the plaque, monocytes differentiate and proliferate into distinct macrophage subtypes. Notably, Ly6C^high^ monocytes predominantly differentiate into M1 macrophages, whereas the prospect of Ly6C^low^ monocytes remains unclear.^(14)^ Macrophages drive chronic vascular inflammation by releasing pro-inflammatory cytokines (interleukin (IL)-1, IL-6, and tumor necrosis factor), similar to the M1 phenotype; whereas anti-inflammatory macrophages secrete IL-10 and transforming growth factor-beta, similar to the M2 phenotype.^(15)^

Foam cells, an early atherosclerosis marker, are characterized by lipid phagocytosis and metabolic functions. However, their death releases lipids and tissue factors, contributing to the formation of the necrotic core, a crucial component of unstable plaques that promotes rupture and thrombosis, causing myocardial infarction.^(16)^ Additionally, macrophages degrade the ECM via matrix metalloproteinases (MMPs), weakening the vessel wall and contributing to adverse remodeling.^(17)^

Beyond M1/M2 macrophages, atherosclerotic plaques harbor novel subtypes, including Mox, M4, Mhem, and M(Hb). Mox macrophages, derived from the bone marrow, exhibit reduced M1/M2 gene expression, enabling heme detoxification, oxidative stress reduction, and foam cell inhibition. Conversely, M4 macrophages, abundant in unstable plaques, express chemokines (CCL2 and CXCL4) and proteases (MMP-12) that facilitate monocyte and neutrophil recruitment, causing ECM degradation. At hemorrhagic sites, M(Hb) and Mhem macrophages regulate erythrocyte turnover. Mhem macrophages, characterized by high CD163 and HO-1 expression, recycle iron and heme, whereas M(Hb) macrophages expressing CD206 and CD163 scavenge free hemoglobin, mitigating oxidative damage.^(18)^

**Figure 1:**
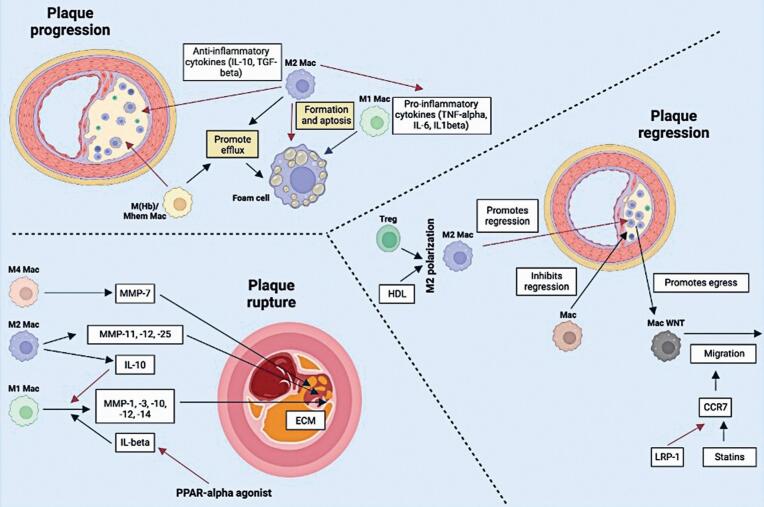
Distinct regulatory mechanisms of macrophage phenotypes in atherosclerosis

These findings offer valuable insights into the complex regulatory mechanisms of macrophages in the progression and regression of atherosclerosis and plaque rupture (Figure 1).^(19,20)^

Plaque progression: Foam cells contribute to plaque progression by binding and internalizing circulating lipids. However, they also facilitate cholesterol efflux, counteracting plaque development. M1 macrophages promote foam cell formation primarily through the secretion of pro-inflammatory cytokines (e.g., tumor necrosis factor (TNF), interleukin (IL)-6, and IL-1), whereas M2 macrophages secrete anti-inflammatory cytokines (e.g., IL-10 and transforming growth factor-beta [TGF-β]), which inhibit inflammation, reduce foam cell formation, and enhance cholesterol efflux. Additionally, M(Hb) and Mhem macrophages mediate cholesterol efflux. Plaque rupture: Macrophage-derived matrix metalloproteinases (MMPs) degrade extracellular matrix (ECM) proteins such as collagen and elastin, weakening the fibrous cap and increasing the risk of plaque rupture. M2 macrophages mitigate this process by inhibiting MMP production through IL-10 secretion. PPARα agonists reportedly suppress IL-1β-induced MMP-12 expression, offering potential therapeutic benefits. *Plaque regression*: During plaque regression, Wnt signaling is activated in macrophages to facilitate migration. Statins and LRP-1 deficiency promote regression by activating CCR7-dependent macrophage migration. M2 macrophages are crucial drivers of plaque regression, with Tregs and HDL promoting M2 polarization (18). Dark blue arrows denote activation, and red arrows indicate inhibition

Source: Created with BioRender. Available from: https://www.biorender.com/

Despite substantial enhancement, the pathogenesis of atherosclerosis remains incompletely understood owing to its complexity, which involves diverse cell types and intricate molecular interactions. Single-cell RNA sequencing (scRNA-seq) overcomes these limitations by providing unprecedented resolution of cellular composition and gene expression profiles. In contrast to bulk RNA sequencing, which involves averaging RNA expression across all cells in a sample, scRNA-seq captures RNA expression at the single-cell level, enabling the identification of cell-type-specific expression patterns and revealing cellular heterogeneity within tissues.^(21)^

**Figure 2:**
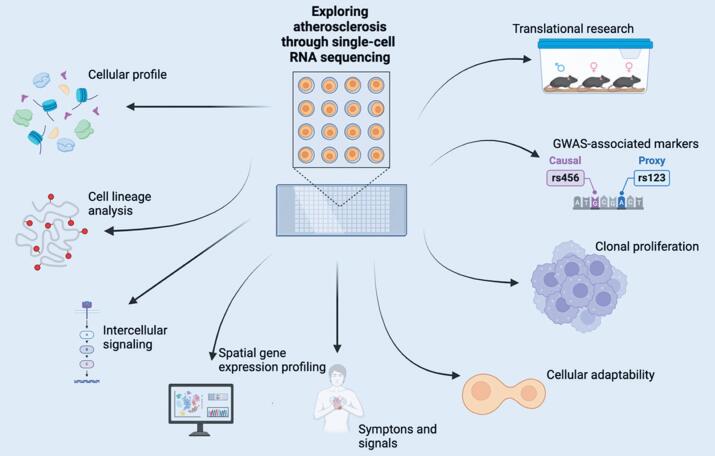
Exploring atherosclerosis through single-cell RNA sequencing

scRNA-seq has provided novel insights into the pathogenesis of atherosclerosis, offering valuable opportunities for therapeutic innovation. This review highlights these advancements and explores their potential impact on understanding and managing atherosclerosis (Figure 2).^(22,23)^

Single-cell RNA sequencing offers a powerful toolkit for exploring various aspects of atherosclerotic disease. scRNA-seq provides valuable insights into lineage tracing, cellular plasticity, and clonal expansion by revealing the cellular composition of atherosclerotic plaques. Additionally, it enables the identification of cell-gene associations derived from markers associated with the genome-wide association study, which are crucial for identifying genetic variants that are vital for understanding disease mechanisms, targeting therapies and precision medicine^(23)^

Source: Created with BioRender. Available from: https://www.biorender.com/

### Investigating cellular heterogeneity in atherosclerosis

Understanding the cellular diversity within atherosclerotic plaques has been hindered by the inherent heterogeneity of the tissue. Histological studies have shown that plaques predominantly comprise VSMCs and infiltrating immune cells. Although advances in omics techniques, such as DNA microarrays and bulk RNA sequencing, have provided valuable insights into the proteomic and transcriptomic profiles of plaque cells, these bulk methods aggregate data from entire cell populations, obscuring the distinct contributions of individual cell types.^(24,25)^

scRNA-seq overcomes this limitation by enabling the identification of known and novel cell populations and revealing subtle transcriptomic variations influenced by specific microenvironments. This breakthrough has driven the development of advanced bioinformatics tools, facilitating in-depth analyses such as trajectory mapping, RNA velocity, and cell-cell communication studies.^(26)^

Future directions include the integration of spatial transcriptomics to provide spatial context to single-cell data, combining genetic and transcriptomic analyses for a comprehensive understanding of atherosclerotic plaque biology, and translating findings from animal models to human studies to enhance clinical relevance.^(27)^

### Advancing the understanding of cellular composition: Insights from scRNA-seq

scRNA-seq has enhanced the understanding of atherosclerotic plaques by providing detailed insights into cellular heterogeneity. This technique has revealed diverse subpopulations of VSMCs, ECs, and immune cells, each exhibiting distinct phenotypes and functional roles. These cellular states critically determine plaque stability and clinical outcomes.^(28)^

Vascular smooth muscle cells play a pivotal role in maintaining structural integrity and regulating blood flow. In atherosclerosis, smooth muscle cells (SMCs) undergo phenotypic modulation, adopting states that influence plaque stability.^(29)^ Human plaques harbor contractile and synthetic SMC phenotypes, with the latter contributing to fibrous cap formation. Emerging evidence suggests the existence of intermediate phenotypes, such as fibrochondrocytes. Similarly, scRNA-seq has revealed the role of TCF21 in mediating SMC transitions, underscoring its significance in fibrous cap stabilization.^(30)^

Endothelial cells form the critical interface between the bloodstream and arterial wall. Additionally, scRNA-seq studies have revealed that EC subsets participate in processes such as angiogenesis and endothelial-to-mesenchymal transition (EndoMT).^(31,32)^ Notably, EndoMT alters SMC composition and contributes to plaque destabilization, although its precise role in disease progression remains under investigation.^(33)^

Immune cell accumulation is a hallmark of atherosclerosis.^(34)^ scRNA-seq has delineated macrophage subpopulations, including resident-like, inflammatory, interferon-inducible, and cavity macrophages.^(35)^ Data from scRNA-seq have superseded those of the initial oversimplified M1/M2 dichotomy.^(36)^ Similarly, T cells exhibit significant heterogeneity, with mixed Th1 and Treg transcriptional profiles that reflect the complexity of immune regulation in atherosclerosis.^(37)^

### Hemodynamics and transcriptomic insights via scRNA-seq

Hemodynamics plays a crucial role in the development of atherosclerosis, with lesions predominantly forming in regions of disturbed blood flow, such as arterial branches and curvatures.^(38)^ The impact of disturbed flow on arterial cell behavior is well-documented, and it promotes atherosclerosis by modulating gene expression in ECs. Insights from scRNA-seq strongly indicate that disturbed flow reprograms ECs, shifting their phenotype from atheroprotective to proatherogenic.^(39)^

### Regional variability in atherosclerotic lesions

Atherosclerotic plaques develop across various arterial regions, including the coronary, carotid, cerebral, aortic, iliac, and femoral arteries. Among these, the coronary and carotid arteries are the most frequently investigated in human studies.^(40)^ Lesion location critically determines plaque morphology and function, influencing the clinical presentation and outcomes of ischemic events.^(41)^ For example, coronary artery plaques result in local thrombosis owing to rupture or erosion, causing downstream tissue ischemia. In contrast, plaques in the carotid circulation frequently cause ischemic damage through embolization,^(42)^ obstructing cerebral arteries. Understanding these site-specific pathophysiological mechanisms is crucial for developing targeted diagnostic and therapeutic strategies.

### Role of single-cell RNA sequencing in precision medicine for atherosclerosis

**Figure 3:**
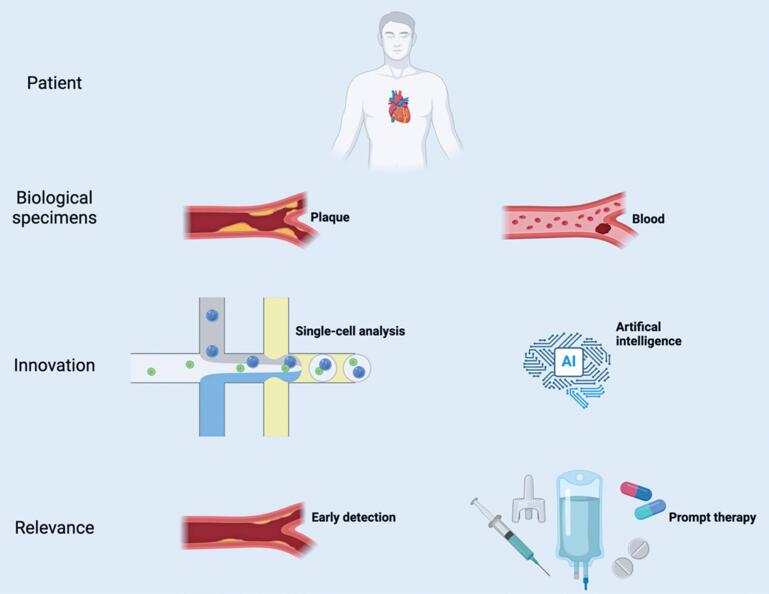
Integrative framework of single-cell analysis in precision medicine for atherosclerosis

Studies on the human genome and disease mechanisms have traditionally focused on the tissue level. However, the advent of scRNA-seq, with its ability to provide high-resolution insights at the cellular level, has significantly advanced the field of precision medicine. Regarding atherosclerosis, scRNA-seq has revealed potential therapeutic targets and facilitated drug discovery, fostering tailored clinical interventions (Figure 3).^(43,44)^

The integration of single-cell analysis and artificial intelligence facilitates a comprehensive examination of tissue heterogeneity within atherosclerotic plaques and in the bloodstream. This multidisciplinary approach enhances the understanding of the pathophysiological mechanisms underlying atherosclerosis and supports the advancement of early diagnostic and targeted therapeutic strategies. Therefore, this strategy was designed to refine patient-specific treatment, which is consistent with the objectives of precision medicine in the effective management of atherosclerosis^(15)^

Source: Created with BioRender. Available from: https://www.biorender.com/

### Early diagnosis of atherosclerosis: Advances and challenges

The early clinical manifestations of atherosclerosis are usually subtle and easily overlooked, as the disease can progress asymptomatically for years before causing overt symptoms.^(45)^ Early and accurate diagnosis is critical for reducing complications and mortality, as it plays a pivotal role in minimizing cardio-cerebrovascular events. Current diagnostic approaches include blood biochemical tests (to identify risk factors) and imaging techniques such as ultrasonography, coronary artery calcium scanning, computed tomography, and invasive coronary angiography (to assess disease scope and severity). However, these diagnostic modalities usually impose a significant economic burden.^(46)^

Early and accurate atherosclerosis diagnosis is crucial for reducing complications and mortality. High-resolution imaging techniques, including photon-counting computed tomography angiography, combined with molecular-level analysis, are promising for improving early diagnosis and preventing cardiovascular events.^(47)^ Ultra-high resolution computed tomography provides a detailed assessment of coronary vessels, including the coronary artery lumen, vascular walls, and atherosclerotic plaques, with superior spatial resolution, an essential feature for early atherosclerosis detection.^(48)^ Additionally, this imaging modality can provide information regarding local inflammation through the analysis of pericoronary fat tissue (the Fat Attenuation Index), validated to predict outcomes.^(49)^

Conversely, single-cell analysis may represent a minimally invasive approach for early atherosclerosis diagnosis.^(50)^ Single-cell transcriptomics involves examining the gene expression level of individual cells in a given population by simultaneously measuring the RNA concentration of hundreds to thousands of genes. Whole-blood gene expression profiling provides valuable insights into atherosclerosis dynamics, potentially revealing pathogenic mechanisms. Several prevalent conditions, including acute MI^(51)^ and different forms of atherosclerosis^(52)^ exhibit distinct gene expression signatures. Analyzing differential gene expressions in peripheral blood cells offers a more comprehensive understanding of disease progression, enhancing the prediction of cardiovascular events beyond conventional diagnostic methods.^(53)^ Notably, alterations in gene expression within peripheral blood cells demonstrate high sensitivity and specificity for diagnosing atherosclerotic coronary artery disease.^(54)^ For instance, the ADIR2 gene expression levels have been associated with the progression of coronary atherosclerosis.^(55)^ Additionally, Meng et al. identified ABCB1, ACSL1, ZHHC9, and other genes as crucial contributors to atherosclerosis pathogenesis.^(56)^ Furthermore, a recent study has revealed that SVEP1 can drive atherosclerosis independently of cholesterol levels.^(57)^ Consequently, single-cell analysis is becoming increasingly attractive for the precise and early diagnosis of atherosclerosis.^(45)^

### Advancements in early atherosclerosis treatment

Effective management of atherosclerosis requires an in-depth understanding of various treatment modalities and their therapeutic outcomes. Clinicians should be well-versed in treatment nuances to make informed decisions. Single-cell analysis may be a valuable tool in this endeavor, offering detailed insights into the efficacy of treatment strategies at the cellular level, which is crucial for optimizing early intervention.^(58)^

Single-cell analysis allows for a detailed evaluation of therapeutic impacts by monitoring changes in cellular composition and gene expression before and after treatment within the same individual or across different groups.^(59)^ For instance, the effectiveness of desmosterol, an immunomodulator targeting lipid dysregulation in atherosclerosis, has been demonstrated through blood single-cell analysis. This method revealed significant changes in the expression of anti-inflammatory macrophage markers post-treatment, affirming its role in mitigating inflammation by modulating macrophage cholesterol metabolism and their activation state.^(60)^ Additionally, RNA-seq analyses of macrophage phenotypes and inflammation-related genes in experimental atherosclerosis have revealed the pharmacokinetic benefits of genistein, enhancing its profile as a treatment option.^(61)^ These findings underscore the utility of single-cell technologies in providing a nuanced assessment of treatment efficacy at the molecular level.

### Current challenges and emerging opportunities in scRNA-seq for atherosclerotic studies

scRNA-seq is promising for elucidating the cellular and molecular mechanisms underlying atherosclerosis, revealing potential pathways for innovative diagnostic and therapeutic strategies. However, the approach is limited by several challenges that should be overcome to fully harness its potential.

### Key challenges

Sample preparation and quality: The calcified and fibrotic nature of atherosclerotic plaques presents significant challenges for cell dissociation. Enzymatic digestion processes risk selectively losing or damaging specific cell populations, and the limited availability of fresh human tissue restricts the generalizability of the findings.^(62)^

Cell population representation: Technical limitations in capturing low-abundance populations can cause underrepresentation of rare cell types, such as immune cell subsets or progenitor cells. Dropout effects and batch variability further hinder the accurate detection of gene expression in small cell subsets.^(63)^

Data analysis complexities: The cellular heterogeneity and dynamic states within atherosclerotic plaques complicate clustering and trajectory analysis. Integrating scRNA-seq data with other multi-omics approaches, such as proteomics or spatial transcriptomics, requires sophisticated computational pipelines. Additionally, interpreting non-coding RNA functions and alternative splicing events remains challenging.^(21)^

Reproducibility and standardization: Variability in scRNA-seq protocols and platforms contributes to inconsistencies across studies. The lack of standardized reference datasets for atherosclerosis hinders cross-study comparisons and limits meta-analysis potential.^(64)^

Translational challenges: Species-specific differences in atherosclerotic plaque composition and progression complicate the translation of findings from animal models to humans. Moreover, *in vivo* functional validation of identified pathways and cell types is resource-intensive and time-consuming.^(65)^

### Opportunities for advancement

Integration with spatial technologies: Combining scRNA-seq with spatial transcriptomics can provide a spatially resolved view of cellular heterogeneity in proliferative atherosclerosis, revealing cell-cell interactions and microenvironmental dynamics.^(66)^

Advances in multi-omics approaches: Incorporating epigenomics, proteomics, and metabolomics will facilitate a more comprehensive understanding of cellular functions. Single-cell multi-omics platforms, such as CITE-seq and scATAC-seq, can reveal gene regulatory networks and lineage trajectories.^(21)^

Enhanced computational tools: Developing robust algorithms to analyze complex datasets, address batch effects, and enable longitudinal data integration will improve data reliability. Machine learning approaches can enhance the discovery of novel cell types and the development of predictive models for atherosclerosis progression.^(67)^

Improved sample accessibility: Innovations in biobanking and single-cell cryopreservation will increase the availability of high-quality human samples. Advanced protocols capable of preserving rare cell types and capturing calcified tissue components will improve cell representation in studies.^(68)^

Focus on clinical translation: Integrating scRNA-seq findings with imaging and clinical data can reveal biomarkers of atherosclerotic plaque stability and disease progression. Targeting crucial cell types or pathways revealed through scRNA-seq can inform the development of precision therapies.^(69)^

Longitudinal and functional studies: Longitudinal scRNA-seq studies can be used to track cellular dynamics during atherosclerosis progression and regression in response to treatment. Functional studies validating the roles of specific cell types and pathways will facilitate the translation of discoveries into therapeutic applications.^(70)^

Cross-species comparisons: Identifying conserved cellular and molecular signatures between human and animal models will enhance the translational relevance of preclinical studies regarding coronary artery disease.^(71)^

## CONCLUSION

scRNA-seq highlights the intricate complexity of atherosclerotic plaques, significantly advancing the understanding of their biology and enabling the identification of novel therapeutic targets. The continued integration of single-cell technologies with advanced computational approaches will further elucidate the molecular mechanisms underlying atherosclerosis, contributing to improved clinical outcomes for patients with cardiovascular diseases.
